# Periodontitis Home Screening with Mouth Rinse Cut-Off 20 Ng/mL aMMP-8 Test and Mobile Application

**DOI:** 10.3390/diagnostics15030296

**Published:** 2025-01-27

**Authors:** Miika Penttala, Timo Sorsa, Julie Toby Thomas, Andreas Grigoriadis, Dimitra Sakellari, Shipra Gupta, Pirjo Pärnänen, Tommi Pätilä, Ismo T. Räisänen

**Affiliations:** 1 Department of Oral and Maxillofacial Diseases, Head and Neck Center, University of Helsinki and Helsinki University Hospital, 00290 Helsinki, Finland; timo.sorsa@helsinki.fi (T.S.); drthomastoby@gmail.com (J.T.T.); pirjo.parnanen@helsinki.fi (P.P.); ismo.raisanen@helsinki.fi (I.T.R.); 2 Division of Oral Diseases, Department of Dental Medicine, Karolinska Institutet, 171 77 Stockholm, Sweden; 3 Department of Preventive Dentistry, Periodontology and Implant Biology, Faculty of Health Sciences, Dental School, Aristotle University of Thessaloniki, 541 24 Thessaloniki, Greece; andreasgrigor@gmail.com (A.G.); dimisak@gmail.com (D.S.); 4 Dental Sector, 424 General Military Training Hospital, 564 29 Thessaloniki, Greece; 5 Oral Health Sciences Centre, Post Graduate Institute of Medical Education & Research, Chandigarh 160012, India; shipra1472@gmail.com; 6 Department of Pediatric Surgery, New Children’s Hospital, University of Helsinki and Helsinki University Hospital, 00290 Helsinki, Finland; tommi.patila@hus.fi

**Keywords:** periodontitis, POCT, aMMP-8, logistic regression

## Abstract

**Background:** In this study, we describe a method by which a patient can independently assess their own periodontitis risk, for example, at home, with a mobile application. The aim of the study is to use active matrix metalloproteinase aMMP-8 mouth rinse cut-off 20 ng/mL point-of-care testing (POCT) and a polynomial function to reveal patients’ statistical risk of periodontitis. **Methods:** The polynomial function presented in this study was modeled with multiple logistic regression and the function estimates the risk of periodontitis using a probability measure. To investigate variables associated with periodontitis, we used data from adult patients visiting dental clinics in Thessaloniki, Greece. **Results:** The research results revealed that with appropriate information it is possible to obtain sufficient accuracy about a patient’s potential risk of periodontitis. The function for estimating risk of periodontitis is PERIORISK = (1 + e^−(3.392×X1+0.002×X2+1.858×X3−9.151)^)^−1^, where X1 = aMMP-8 test result and tobacco smoking status, X2 = age × waist circumference and X3 = patient’s individual and parental history of diabetes. **Conclusions:** The prediction of periodontitis risk using an aMMP-8 test and a polynomial function seems to be a useful, non-invasive, safe-to-use and cost-effective tool for all people. Overall, in the model created, mouth rinse cut-off 20 ng/mL aMMP-8 test result, age, waist circumference, tobacco smoking status and patient’s individual and parental history of diabetes were found to be good factors explaining the risk of periodontitis.

## 1. Introduction

Periodontitis is a common chronic non-communicable disease and is associated with many conditions, e.g., diabetes and cardiovascular diseases [1,2]. According to data from 2009 to 2014, the prevalence of some category of periodontitis among dentate US adults aged 30–79 years was 42.2% [3]. Furthermore, between 2011 and 2020, the prevalence of periodontitis in dentate adults was estimated to be approximately 62% worldwide [4]. The need for methods for detecting periodontitis is obvious. The method presented in this study to determine the risk of periodontitis is non-invasive and cost-effective, and it is easy to use, for example, at home.

To investigate periodontitis, we used results from Greece from the study Active MMP-8 (aMMP-8) as a Grading and Staging Biomarker in the Periodontitis Classification [5]. The results of the study included that the aMMP-8 point-of-care mouth rinse test can be utilized as an adjunctive and preventive diagnostic tool and biomarker to identify periodontal disease, classified by stage and grade, and ongoing periodontal breakdown chairside in clinical practice in only 5 min. It has been known since the 1990s that aMMP-8, but not total MMP-8, is an indicator of irreversible collagenolytic periodontal tissue destruction [6,7,8,9,10]. The aim of the present study was to find a polynomial function screening for periodontitis with the help of aMMP-8 mouth rinse cut-off 20 ng/mL point-of-care testing (POCT).

## 2. Methods

### 2.1. Framework

We propose a framework to build a prediction model to facilitate the diagnosis of periodontitis. The framework includes multiple logistic regression analysis to create a function with specific variables to estimate a patient’s risk of periodontitis. Patients who are considered to be at risk of periodontitis are referred to a dentist for follow-up.

### 2.2. Sample Data and Parameters

The dataset used in this study was collected from adult Greek patients who visited the Department of Periodontology, Dental School, Aristotle University, Thessaloniki, Greece, and the Periodontal Department of 424 General Army Hospital, Thessaloniki, Greece. Participants signed an informed consent form and the study was conducted according to the protocol outlined by the Research Committee of the Aristotle University of Thessaloniki, Greece, and approved by the Ethical Committee of the School of Dentistry (protocol number #64, 12 June 2018). All procedures performed in the present study involving human participants were in accordance with the ethical standards of the institutional and/or national research committee and with the 1964 Helsinki declaration and its later amendments, or comparable ethical standards [11,12].

The inclusion criteria in the study were as follows: age of patients ≥ 18 years (men or women), the presence of at least 20 teeth, patient physical status 1 or 2 in A.S.A. classes according to the classification of American Society of Anesthesiology, the written informed consent of the research participants and the result of the self-assessed questionnaire proposed by the Centers for Disease Control and Prevention (CDC, Atlanta, GA, USA), for patients at high risk of developing Diabetes Mellitus) ≥9. The exclusion criteria in the study were age of patients < 18 years, the presence of diabetes mellitus or immunomodulatory diseases, a medication intake that affects glycemic control, periodontal therapy for the last 6 months and women in pregnancy or lactation.

The patients were selected by result of the self-assessed questionnaire for patients at a high risk of developing diabetes mellitus. In the sample data, 4.7% had diabetes (HbA1c ≥ 6.5%) and 16.2% had a condition called prediabetes (5.7% ≤ HbA1c < 6.5%), which adequately represented the normal prevalence of the diseases aforementioned in the Greek population [13]. The HbA1c levels were analyzed from the HbA1c blood test results. The patient sample data were found to be suitable for this study, and the study’s data association with diabetes and prediabetes screening had no impact on the validity of the sample data.

Several parameters including, e.g., age, weight, height and waist circumference were recorded. Periodontal examination included clinical measurement of, e.g., probing depth (PD), clinical attachment Level (CAL), bleeding on probing (BOP) and Plaque Levels on six surfaces of each tooth, excluding third molars, using an automated probe (Florida Probe, Florida Probe Corporation, Gainesville, FL, USA) by one calibrated examiner. The study was conducted in 2017–2018. No missing data issues were to be reported in this study. In addition, we followed the STROBE statement to report our cross-sectional observational study.

The patients were classified according to the 2018 classification of periodontal diseases [14,15] and, for example, assessments of bone loss, attachment loss and probing depth were criteria for determining the stages and grades of periodontitis. The periodontal probing depth was measured from the gingival margin to the base of the periodontal pocket or sulcus. The extent of CAL (periodontal tissue destruction) was assessed by calculating the distance from the cemento-enamel junction (CEJ) to the base of the pocket/sulcus. The proportions of the staging and grading classification results are presented in Table 1. Patients’ specific probing depth data for each stage of periodontitis and all data related to the creation of the model introduced in this study are directly from the original research published [11].

Table 1 presents the essential data of the study. Waist circumference was measured between the top of the hip bone and the bottom of the ribs. A measuring tape was placed halfway between these points and wrapped around the waist loosely enough to fit one finger inside the tape (with the patient exhaling normally). The patients’ diabetes status was determined with an HbA1c blood test (HbA1c ≥ 6.5%) and the history of diabetes in the family was recorded according to the patients’ report. The patients’ tobacco smoking status was determined by the question smoker or non-smoker.

### 2.3. Point-of-Care Testing (POCT)

This research’s idea of creating a polynomial function is based on the active matrix metalloproteinase aMMP-8-utilized point-of-care testing (POCT). The aMMP-8 chairside/point-of-care (PoC) oral fluid tests have continuously been successful in identifying active periodontal tissue destruction and active periodontal disease (periodontitis, peri-implantitis, subclinical periodontitis) among adults and adolescents of different ethnic populations [16,17,18,19]. Furthermore, the impact of the aMMP-8 test result, positive or negative, on the polynomial to be created was investigated in the regression analysis. The aMMP-8 test result was considered positive if the aMMP-8 level of concentration of the mouth rinse test was found to be ≥20 ng/mL, and any other result was marked as a negative test result.

Matrix metalloproteinase (MMP)-8 (neutrophil collagenase-2) levels in its active form (aMMP-8) in the collected oral rinses were analyzed quantitatively by the chair-side/PoCPerioSafe^®^ (Medix Biochemica Ltd., Espoo, Finland) immunotest accompanied by the digital reader ORALyzer^®^ (Dentognostics GmbH, Solingen, Germany)according to the manufacturer’s instructions [11]. The aMMP-8 test was administered to the participants by a trained and calibrated investigator (AG). Briefly, there was a 30 s pre-rinsing with tap water and a one minute wait after the pre-rinse before collecting the mouth rinse sample after 30 s of rinsing with 5 mL of test solution. Three drops of the sample solution were used in the test system, and the result was read on the digital reader within 5–6 min. According to the manufacturer’s recommendation, the test result was considered positive if the aMMP-8 concentration was ≥20 ng/mL. The aforementioned idea of the test was performed as described by Räisänen et al. [20].

### 2.4. Periodontitis Classification

Further on in this study, patients with periodontitis were recorded with a binary value of one, and patients without evidence of periodontitis were recorded with a zero value. All patients were classified by the 2018 classification of periodontal diseases, as mentioned earlier in the study [14,15].

### 2.5. Logistic Regression and Assumptions

The modeling of polynomial functions predicting periodontitis was performed by using multiple logistic regression, and the variables were chosen by a backward stepwise method [21,22,23,24,25,26]. Because the function was developed for home-use, variables which needed dental expertise were excluded. Possible variables for the function to be created were, among others, age, sex, weight, height, waist circumference, tobacco smoking and the result of the aMMP-8 test (cut-off 20 ng/mL). Variable candidates also included combinations such as body mass divided by the square of the body height (BMI) and waist circumference divided by height (waist to height ratio) or, e.g., age multiplied by waist circumference.

The linearity assumption in logistic regression was investigated from scatterplots. In addition, autocorrelation was investigated by the Durbin–Watson test to ensure that the residuals were independent of each other. If this assumption was violated, the significance results of coefficients could be inflated, leading to an incorrectly specified prediction model. Further on, multicollinearity was checked with the variance inflation factor (VIF). Strong multicollinearity increases the variance of a regression coefficient which leads to more inaccurate estimates of the coefficients of the variables. A typical example of strong multicollinearity is detected when at least two predictor variables are highly correlated to each other. To check for multicollinearity and autocorrelation, multiple linear regression was performed with the same dataset mentioned earlier. It was also agreed that samples with a Cook’s distance greater than 0.5 or standardized residuals greater than ±3.0 would be investigated more closely than usual.

### 2.6. Sample Size

In this study, the papers of Peduzzi et al. [27] and Long [28] were used to determine if the sample size was sufficient to model the generated polynomial function. Peduzzi et al. suggest that a number of events per variable (EPV) under 10 could lead to major problems. (Number of events = lower total number of possible outcomes and, i.e., in this study the number of patients without evidence of periodontitis.) We set the criterion that the EPV must be at least 10. Furthermore, Long suggests that multiple logistic regression, as in our study, should not be performed if the sample size is less than 100, and we set this number as an absolute lower limit for how many samples should be in the data.

### 2.7. Statistical Analysis

Sensitivity and specificity were important criteria when choosing the variables of the polynomial function presented in this study. In addition, accuracy and F1 score were used to investigate the results of the polynomial function. The Chi-square goodness of fit test between the observed and predicted binary values was calculated for the results of this study. Also, the phi coefficient φ for categorical data was calculated to investigate the correlation between observed and estimated binary values. A phi coefficient of φ = 0 would indicate no relationship between the binary variables, and the further away from 0 the value of φ would be, the better it would support the possible finding that the model explains the observed data to an acceptable level. The receiver operating characteristic curve (ROC curve) was used to determine the optimal cut-off value for the presence or absence of periodontitis, and the area under the ROC curve (AUC) was also calculated. All the variables in the polynomial function created were agreed to have a significance level of *p* below 0.05. The commercial IBM SPSS Statistics (version 29) computer program was applied in this method.

## 3. Results

The number of patients investigated in this research was 149, including 31 patients with no indication of periodontitis and 118 patients with periodontitis. As written later, one sample was removed from the dataset as an outlier. The final sample size was 148, including 30 patients without periodontitis (number of events = 30). The polynomial function presented in this study had three independent variables (Table 2). It follows that the events per variable (EPV) in our study was exactly 10, and since Peruzzi et al. suggests that the number of EPVs should be at least 10, it can be shown that this criterion was met in this study. Furthermore, Long suggests that multiple logistic regression should not be performed if the sample size is less than 100, and this criterion was also met since the sample data contained 148 observations. Therefore, the sample size forming the polynomial function presented in this study was agreed to be sufficient based on the papers of Peruzzi et al. and Long [27,28].

The linearity assumption was investigated from scatterplots. The predicted log-odds and the values of the continuous explanatory variable were found to have a linear relationship. Multicollinearity was checked with VIF, and multiple linear regression found VIF values between 1.014 and 1.019, indicating no problematic correlation between independent variables. The Durbin–Watson score was calculated, and the resulting score of 1.814 revealed no evidence of autocorrelation. From the continuous independent variable, no values were found to exceed +3 standard deviations from the mean. One sample was investigated more carefully because it had a Cook’s distance value above 0.5 (i.e., 0.7) and a standardized residual more than 3 (i.e., −5.5). After a careful review of all the residuals, the sample mentioned was removed as an outlier from the sample data. The model and its variables were the same with or without the outlier, but some differences were found in the beta coefficients which lead us to remove the outlier. Without the outlier, the model sensitivity, specificity and accuracy were 94.1, 76.7 and 90.5, respectively, and with the outlier, the sensitivity, specificity and accuracy were 94.1, 74.2 and 89.9, respectively.

**Figure 1:**
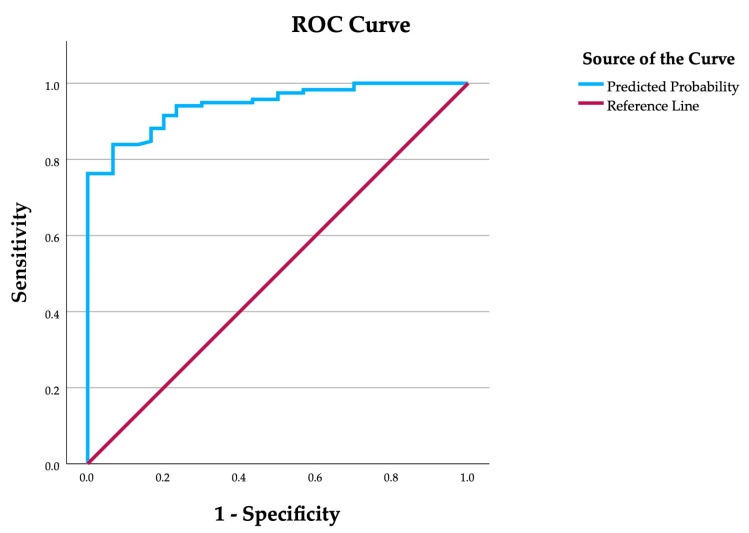
The receiver operating characteristic curve (ROC curve) was used to determine the optimal cut-off value (i.e., 0.55) for a possible periodontitis risk. The area under the ROC curve (AUC) was found to be 0.944 (95% CI = 0.909–0.979).

Pearson’s chi-square goodness-of-fit test between the observed and predicted binary values was investigated, and the result revealed a sufficient goodness of fit of the model: χ^2^ (1, N = 148) = 0, the *p* value equals 1. The phi coefficient φ for the categorical data indicated that the correlation between the observed and estimated binary values was φ = 0.71 and this supported the conclusion that the model fit the data to an acceptable level. The ROC curve was used for determining the optimal cut-off value for the presence or absence of periodontitis, and the cut-off value was determined to be 0.55 (Figure 1).

**Figure 2:**
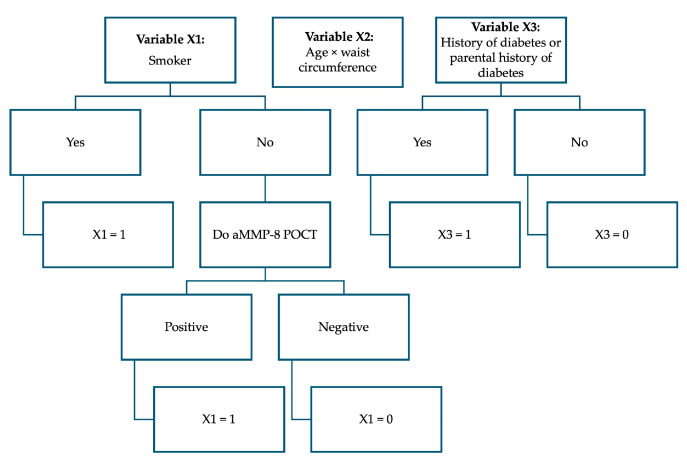
Flowchart to determine variable values.

As a result of this study, in the polynomial function created from the Greek dataset, the best variables describing periodontitis risk were X1 = aMMP-8 POCT outcome and tobacco smoking status, X2 = age multiplied by waist circumference and X3 = patient’s individual and parental history of diabetes. The coefficient output data of multiple logistic regression modeling is presented in Table 2, and Figure 2 shows a flowchart for determining variable values.

The polynomial function created in this study to estimate the risk of periodontitis is as follows:

$$PERIORISK\;=\;\frac{1}{1+e^{-\left(3.392\times X1+0.002\times X2+1.858\times X3-9.151\right)}}$$

**Figure 3:**
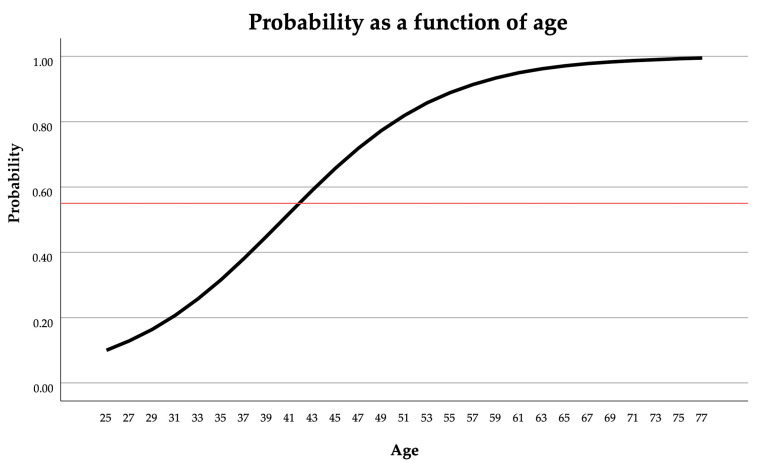
The probability of periodontitis risk as a function of age. Patient data in the example is as follows: positive aMMP-8 test result or tobacco smoker, waist circumference 80 cm and patient’s individual and parental history of diabetes is negative. The probability cut-off value is 0.55 (red line in the figure). The patient has an increased risk of periodontitis if the *x*-axis value is 41.9 years or more.

In which:

PERIORISK = the probability of periodontitis risk [No.] (if the result ≥ 0.55, the patient is statistically at risk of periodontitis; otherwise, the patient is not statistically considered to have periodontitis risk (Figure 3)).

X1 = a positive aMMP-8 POCT outcome or a tobacco smoker [No.] (yes = 1, a negative aMMP-8 POCT outcome = 0, a non-smoker must take the aMMP-8 test).

X2 = age × waist circumference [yrs. × cm] (possible input 2200–8772, patient boundary conditions are as follows: 25 yrs. ≤ age ≤ 78 yrs., WC 60 cm–152 cm).

X3 = patient is diabetic or patient’s parent is diabetic [No.] (yes = 1, otherwise = 0).

The polynomial function presented in this study detected 94% (111/118) of periodontitis patients from the sample size it was originally modeled on. The polynomial function found 7/30 false positives and therefore the proportion of true negatives was 77% (23/30) of the same previously mentioned sample size. Therefore, one can suggest that the polynomial function, created in this study, has a sensitivity of 94.1% (95% CI = 89.8–98.3%) and a specificity of 76.7% (95% CI = 69.0–84.3%) when tested against the patient population it was formed from. The area under the ROC curve (AUC) was found to be 0.944 (95% CI = 0.909–0.979) (Figure 1). The accuracy and F1 score were calculated, and they were 90.5% and 0.941, respectively. The values of accuracy and F1 score were considered to have reached the required level of acceptance and supported the conclusion that the developed polynomial function had achieved sufficient accuracy in this study.

## 4. Discussion

Using the method presented in this study, the patient can independently assess their own risk of periodontitis. Home-use screening for periodontitis is possible due to the aMMP-8 mouth rinse cut-off 20 ng/mL point-of-care-test in connection with the polynomial function. In the future, this combination of a test and a polynomial function is reasonable to combine into an easy-to-use-at-home mobile personalized medicine application.

The data in this study was limited, and additional patient data would potentially increase the accuracy of the generated function, although sensitivity (94%) and specificity (77%) revealed sufficient and encouraging results. For future research, patients should be asked more specific questions about their medical history, diseases and other illnesses, possible malnutrition or, e.g., exercise habits.

The main idea of this study was to find statistical indicators that describe healthy individuals without evidence of periodontitis. The stages and grades of periodontitis are different in their characters, and the focus in this study was on the parameters explaining patient health. The key factor was the ability of an aMMP-8 test, with a cut-off of 20 mg/mL, to find healthy patients with a specificity rate of 93% (28/30 on Table 1) [5,29,30]. Mouth rinse aMMP-8 levels <20 ng/mL are regarded as biomarkers of periodontal health [29,30]. Based on the patient’s tobacco smoking and aMMP-8 test result, a special variable was created to screen the sample data to find healthy patients. A binary value of zero was statistically more likely to indicate a healthy individual, and a value of one indicated the opposite. Based on the regression analysis, it was concluded that a tobacco smoker can use the formula created in this study without the aMMP-8 test. It is well known that tobacco smoking is strongly linked with periodontitis. For example, in a meta-analysis of 14 studies, it was shown that pooled adjusted risk ratios estimated that smoking increases the risk of periodontitis by 85%. Smoking had a detrimental effect on the incidence and progression of periodontitis [31]. In this dataset and sample size, it was not possible to create a single variable for the aMMP-8 test and smoking. This would be the most likely scenario as the sample sizes expand. It is worth mentioning that historical data on smoking, including possible smoking cessation or pack-years smoked, were not available. Also, the number of cigarettes smoked per day by patients who smoked was not available. It is important that these factors are comprehensively investigated in the future development of the polynomial function. In addition, we found no association between tobacco smoking status and aMMP-8 test results (cut-off 20 ng/mL) in this dataset.

Age was an important factor in the function created. However, the regression analysis found that age multiplied by waist circumference was also a good predictor of periodontitis. If the diabetes-related variable was omitted from the function introduced in this study, several different equations were still found to meet the criteria of a good polynomial function. In that case, there were only two variables in the equation. As just one example of the variables, for further development, a binary variable age multiplied by waist circumference with a cut-off of 4800 (unit = yrs. × cm), together with the mentioned aMMP-8/smoking variable would possibly be good variables to research more. One could predict that age combined with possible nutrition and exercise factors may be a good variable for the future development of the function. It is worth mentioning that while epidemiological data are clear that periodontitis increases significantly in prevalence and severity with aging, the fundamental biological mechanisms generating this susceptibility remain ill-defined [32,33,34,35].

Patients’ individual and parental history of diabetes was included in the polynomial function created. Periodontitis and diabetes have a two-way interlinked relationship and a first-degree relative with diabetes is an established risk factor for developing diabetes [1,36]. Epidemiological studies confirm that diabetes is a significant risk factor for periodontitis. In the sample data of this study, the prevalence of diabetes was 4.7% and it was determined by an HbA1c blood test. The study was conducted in the area of Thessaloniki, where the prevalence of diabetes can be estimated at 10% [13]. Variables, including diabetes-related factors, have the potential to increase the accuracy of the polynomial function as datasets grow and diversity increases. Diabetes can be an undiagnosed disease, and this proportion is estimated to be 1.5% among adults in Greece and, for example, in the USA [13,37].

In the future, it will be important to study the effect of medication use on periodontal health more comprehensively. Patients’ current and previous medications should be determined during examinations. In this study, only the intake of medications affecting glucose balance, which were included in the exclusion criteria for participation in the study, could be taken into account. The effects of medications include, e.g., effects on gingival tissues and on salivary flow. Although many medications increase the risk of periodontal disease, a few may decrease the risk [38]. The most interesting medicines to research further would possibly be antidepressants, diabetes medicines and, for example, low-dose doxycycline [39,40,41]. Other subjects to be studied would be nutrition and exercise. A study conducted in Saudi Arabia, for example, found that engaging in the recommended level of exercise and consuming a high-quality diet was associated with a lower risk of periodontitis [42].

The statistical approach of the study was the binary classification of periodontitis status. It was chosen to simplify the statistical risk assessment. In the future of the development of the method, it may be possible to achieve a predictor model that also takes into consideration the staging or grading of periodontitis. Ordinal multinomial logistic regression would possibly suit in this approach as the development of the method continues. In addition, a continuous variable of aMMP-8 concentration in the mouth rinse would possibly also be a suitable predictor for, e.g., grading periodontitis. aMMP-8 concentration has been found to have an association with the stage and grade of periodontitis [5].

In further development of the method introduced in this study, statistical testing is an important part; e.g., cross-validation can be one of the most useful methods. Its main idea is to divide the data into two segments and test for, e.g., possible statistical challenges, for example, overfitting (too-low EPV) by using data from one segment to test the function produced by the other segment. This would allow for the examination of different models and their diagnostic accuracy based on different EPV levels, potentially reducing the risk of overfitting and increasing the generalizability of the model.

The dataset used in this study is from population of Thessalonica area, Greece. It should be noted that the polynomial function created in this study can only be applied to the population mentioned without further research. The findings of this study should also be confirmed in real life, as the model created in this study was only tested with the data from which it was created.

Multiple logistic regression, i.e., supervised machine learning, has been used to study oral health prior to this study, and promising results have been reported when also using a cut-off of 10 ng/mL mouth rinse aMMP-8 as a variable [43]. Notably, Aji et al., Thomas et al. and Gupta et al. have separately shown in Finnish, Greek and Indian cohorts that a cut-off of 20 ng/mL, compared to suggested 10 ng/mL and 25 ng/mL cut-offs, is a more precise periodontitis aMMP-8 biomarker level [10,29,43,44,45,46,47]. Overall, larger sample sizes and more complex predictors of periodontitis are required before machine learning can be leveraged to its full potential for the prediction of periodontitis. In addition, any models that are developed should undergo robust external validation before claims are made regarding their potential impact on the wider population [48].

One can conclude that, at least in theory, the variables presented in this study would be broadly applicable in the future development of the method introduced. For example, the prevalence of periodontitis increases with age in different populations [35,49,50]. Also, the aMMP-8 enzyme and smoking, factors utilized in our prediction model, have also been found to be associated with periodontitis in many ethnic populations [5,31]. The same is suggested by the current literature concerning diabetes [1].

Finally, it is important to emphasize that the home-screening tool presented in this study is intended to complement, rather than replace, traditional clinical diagnostic methods for periodontitis. Traditional diagnostics, such as periodontal probing and radiographic analysis, provide direct clinical evidence of the presence of the disease and require in-person dental visits. However, in the future development of the method, new variables may be possible to add from the area of dental expertise; e.g., estimation of dental plaque % or the number of teeth are factors that should be investigated as a candidate for variables in the function. The method introduced allows individuals to more proactively monitor their statistical probability of having periodontitis. Also, medical doctors and other health professionals can use the tool to identify patients at risk of periodontitis or to possibly find periodontitis-related diseases [1,2].

## 5. Conclusions

According to our knowledge, this is the first time for use at home that a polynomial function has been introduced to non-invasively estimate from oral fluid, i.e., a mouth rinse aMMP-8 test, the risk of having periodontitis. Predicting periodontitis with an aMMP-8 test result (cut-off 20 ng/mL) by using polynomial functions appears to be useful, safe-to-use and a cost-effective personalized medicine tool for all people. This is related to the connection of periodontitis and aMMP-8 concentration in mouth rinse, and aMMP-8 POCT is a convenient way to assess the risk of periodontitis.

## Figures and Tables

**Table 1:** A comparison of the general characteristics in the dataset used to create the polynomial function revealing periodontitis. The data are presented as mean ± SD for continuous variables and as a number for the categorical variables. BOP, bleeding on probing; aMMP-8, active matrix metalloproteinase-8; CAL, clinical attachment level; PPD, periodontal probing depth.

Patient’s Data	Periodontal Status	
	No Evidence of Periodontitis (n = 30)	Stage I, II, III (n = 118)
**Gender**		
Female	11	64
Male	19	54
**Smoking status**		
Tobacco smoker	3	41
Non-smoker	27	77
**Diabetic status**		
Diabetic	0	7
Non-diabetic	30	111
**Parent with diabetes**		
Yes	10	47
No	20	71
**Age category**		
25–49 year olds	24	30
50–78 year olds	6	88
**Age (yrs.)**	42 ± 11	56 ± 10
**Body mass index (kg/m^2^)**	30.6 ± 4.5	30.0 ± 5.0
**Weight (kg)**	93 ± 18	87 ± 18
**Height (cm)**	174 ± 11	170 ± 9
**Waist circumference (cm)**	100 ± 18	103 ± 16
**Waist to height ratio (cm/cm)**	0.57 ± 0.08	0.61 ± 0.09
**aMMP-8 levels**		
aMMP-8 ≥ 20 ng/mL	2	50
aMMP-8 < 20 ng/mL	28	68
**Stage of periodontitis status**		
No evidence of periodontitis	30	0
Stage I	0	14
Stage Il	0	81
Stage III	0	23
**Grade of periodontitis status**		
No evidence of progression	30	0
Grade A	0	14
Grade B	0	90
Grade C	0	14
**CAL (mm)**	2.4 ± 0.45	3.5 ± 1.1
**PPD (mm)**	2.3 ± 0.34	3.1 ± 0.84
**Number of teeth present (No.)**	27 ± 2	24 ± 3
**BOP (%)**	42 ± 26	54 ± 23

**Table 2:** Coefficient output data of multiple logistic regression modeling.

Model	B	S.E.	Wald	df	Sig	Exp (B)
**aMMP-8 test (cut-off 20 ng/mL)/tobacco smoking status**	3.392	0.772	19.309	1	<0.001	29.74
**Age × waist circumference**	0.002	0	20.703	1	<0.001	1.002
**Patient’s individual and parental history of diabetes**	1.858	0.753	6.084	1	0.014	6.411
**Constant**	−9.151	2.072	19.509	1	<0.001	0

## Data Availability

The dataset presented in this article are not readily available and restrictions apply to the dataset due to the technical time limitations of the ongoing study. Requests to access the datasets should be directed to the corresponding author.
